# Cardiovascular disease risk scores in the current practice: which to use in rheumatoid arthritis?

**Published:** 2014

**Authors:** A Purcarea, S Sovaila, A Gheorghe, G Udrea, V Stoica

**Affiliations:** * Strasbourg Medical University; Internal Medicine Department, Civil Hospital, Strasbourg, France; ** Internal Medicine Department, Civil Hospital, Strasbourg, France; *** Internal Medicine Department, Hospital Geomed-Klinik, Gerolzhofen, Germany; **** ”Carol Davila” University of Medicine and Pharmacy; Internal Medicine and Rheumatology Department,“Cantacuzino” Hospital, Bucharest, Romania

**Keywords:** cardiovascular disease, risk scores, rheumatoid arthritis, chronic inflammatory diseases, risk prediction

## Abstract

Cardiovascular disease (CVD) is the highest prevalence disease in the general population (GP) and it accounts for 20 million deaths worldwide each year. Its prevalence is even higher in rheumatoid arthritis. Early detection of subclinical disease is critical and the use of cardiovascular risk prediction models and calculators is widely spread. The impact of such techniques in the GP was previously studied. Despite their common background and similarities, some disagreement exists between most scores and their importance in special high-risk populations like rheumatoid arthritis (RA), having a low level of evidence. The current article aims to single out those predictive models (models) that could be most useful in the care of rheumatoid arthritis patients.

## Introduction

Cardiovascular disease is the highest prevalence disease in the GP and it accounts for 20 million deaths worldwide each year. While people from any category could suffer from cardiovascular disease, it is especially problematic in special patient groups, like those with chronic inflammatory diseases [**1**]. The first line of action against the effects of cardiovascular disease is an early diagnosis and the use of primary prevention measures in high risk populations [**2**]. Following the 1960's Framingham cohort [**3**], a series of risk factors with pathogenic implication for cardiovascular disease were established. Progressively, models of algorithmic prediction of cardiovascular disease based upon combinations of these traditional risk factors were validated [**4**]. Some algorithms were enriched with certain biomarkers or morphological measurement with no direct pathogenic effect but with a verified association with CVD [**5**].

Rheumatoid arthritis characterizes a particular group of patients with specific characteristics in whom the cardiovascular disease burden is higher than the GP [**6**]. Current practice presupposes the addressing of cardiovascular disease risk promptly but there is no prediction model that is clearly adapted for this purpose. EULAR recommendations [**7**], based on a previous meta analysis state that increased CVD risk should be addressed by adding a fixed multiplier of 1.5 to 2 of the established CVD prediction algorithms when patients have at least 2 out of 3 of the following conditions: a long standing diseases (>10 years), with extra-articular disease or seropositivity for RF or ACPAs. Evidence supporting this algorithm in real life is scarce, and both the detection and treatment of CVD remain less than optimal [**8**,**9**].

## Objective

Our review aims to single out the current or potential CVD risk assessment scores, point out their advantages and limitations in their use in rheumatoid arthritis and discuss the possible pitfalls tied to the RA specific risk profile.

## Methods

**Literature Search**

On the 20th of April 2014, we performed (AP and AG) two independent multistep reviews of literature cited in PubMed with release dates starting in 1991 and ending in 2014, using a specific algorithm and selected a number of models of CVD risk prediction. Because the algorithm had proved to have a limited discovery capacity, a Google search and analysis of personal data was performed and supplementary models escaping our initial algorithm were included. In order to correctly characterise the additional models, another PubMed search for names was conducted.

**Eligibility and model selection**

The identified models were assessed and scored for specific criteria: the existence of a derivation and validation cohorts, number of external validations, type of cardiovascular risk assessed, inclusion in national or international guidelines - or notoriety, expression of risk categories, variables used, existence of a verifiable algorithm and availability of a calculator in different formats. In order to respond to rheumatoid arthritis specificities, models using inflammatory or disease specific variables were especially targeted.

The models presented used results derived from secondary prevention or symptomatic patients, animal models or models that suffered recent updates that had no available full text articles, written in a language other than English or French or calibrated for minorities or specific diseases were also excluded. Models in which the lipid profile was substituted with the BMI surrogate were also excluded.

Using the scoreboards and their medical judgement, 3 individual assessors (AP, SS and AG) with clinical experience selected those models.

For each selected study, a standardised form was used to extract representative data: name, number of participants, geographic considerations, number of variables, outcomes measured, discrimination statistics, presence in guidelines, risk stratification categories.

All the independent studies implementing the previously selected models in rheumatoid arthritis were analysed.

We referred to cohort and prospective studies for the derivation/validation of the scores and to longitudinal controlled studies, cohorts and cross sectional studies for the measure of impact in rheumatoid arthritis.

## Results

**Cardiovascular risk prediction models in the GP**

We identified a number of 5381 articles, from which a number of 33 models were extracted. The use of inclusion and exclusion criteria led to the selection of a number of 10 models. After the final analysis by the study investigators, 7 models were reviewed. The derivation cohorts of the models ranged from 8491 to 1.535.583 subjects. Their discrimination for predefined CVD outcomes in the GP expressed as area under the receiver operated curve (AUROC) varied from 0,695 to 0,840. Additional study characteristics are presented in **Table 1**.

**Table 1:** Models of CVD disease prediction

	Framingham Risk Score (FRS) [**10**]	Atherosclerosis in communities (ARIC) [**11**]	Atherosclerosis in communities with c IMT measurement (ARIC IMT) [**12**]	HeartSCORE (SCORE) [**13**]	Reynolds Risk Score (RSS) [**14**,**15**]	QRisk2 [**16**]	PROCAM [**17**]
Derivation study	Framingham cohort	Atherosclerosis in communities cohort	Atherosclerosis in communities cohort	Pooled European cohorts	Pooled cohorts	Prospective nonrandomized database	MONICA cohort
Statistical method	Cox	Cox	Cox	Weibull / Cox	Cox	Cox	Weibull
Discrimination AUROC	0,733 to 0,788	0,695 to 0842	0,755	0,70 to 0,84	0,708 - 0,808	0,792	N/A
Sample size	8491	14054	14054	205178	27124	1535583	26975
Starting year	1968	1987	1987	1972	1992	1993	1978
Number of variables	8	9	10	6	8	14	9
Notoriety	Guideline	No	No	Guideline	No	No	No
Age of participants mean (extremes)	48,5 to 49.1 (30-75)	54 (45 to 64)	54 (45 to 64)	(19-80)	45 +	48,5 (35-75)	45,7 (20-78)
Outcome measured	CVD Hard	CVD	CVD	CVD Mortality	CVD hard	CVD	CVD Hard
RA specific	No	No	No	No	No	Yes	No
Ease of use	Clinical + lab	Clinical + lab	Clinical, lab, ultrasound	Clinical, Lab	Clinical, Lab	Clinical, Lab	Clinical, Lab
Country	US	US	US	Europe	US	UK	Germany
Calculator available	Yes	Yes	Yes	Yes	Yes	Yes	Yes
10 year risk estimates	percentage	percentage	percentage	percentage	percentage	percentage	percentage
Calculator risk categories	<5%, 5- 10%, 10-20%, >20%	<5%, 5- 10%, 10-20%, >20%	<5%, 5- 10%, 10-20%, >20%	<5%, 5-9%, 10-15% > 15%	<5%, 5- 10%, 10-20%, >20%	<5%, 5- 10%, 10-20%, >20%	<10%, 10-20%,>20%
RA Validation	yes	no	no	yes	yes	yes	yes

**Outcomes**

Each model clearly defined its outcomes but some differences exist between studies. FRS explored the occurrence of global CVD events, such as angina or coronary insufficiency and peripheral artery disease. The ARIC cohort with the two models proposed (ARIC and ARIC-IMT) explored CVD events defined by death, MI, coronary revascularisation or proven ECG changes consistent with an ischemic origin. SCORE followed fatal CVD events. For RRS, the CVD disease was defined by hard events like stroke, MI, revascularisation and death. The QRisk2 model CVD outcomes are defined as angina, MI or stroke. PROCAM had outcomes based on CVD events described either by hard events like MI and stroke but also by ECG and cardiac enzymes changes.

**Comparative studies in rheumatoid arthritis**

Two longitudinal and three transversal studies comparing different models of CVD risk prediction were identified. FRS and SCORE were used in one longitudinal comparative study in their EULAR modified form (mFRS and mSCORE). Only one longitudinal study compared FRS with SCORE, Qrisk2 and RRS. The 3 cross-sectional studies cited compared either SCORE with mSCORE (Additional data presented in **Table 3**).

**Table 3:** Models populations

					Performance of RA tested models in longitudinal or transversal studies						
					FRSL	ARIC	ARIC-IMT	PROCAM	SCORE	Raynolds	QRisk
Study author	Year	Design	Outcomes / Duration	Sample size	Study specific discriminates						
Crawson [**18**]	2008	longitudinal	CVD	580	AUROC 0,505 - 0,707 Underestimates risk by 65 - 100% especially in high risk groups	N/A	N/A	N/A	N/A	AUROC N/A Underestimates risk when CRP > 15	N/A
Gomez-Vaquero [**19**]	2014	Cross sectional	Surrogate cIMT with severe lesions: IMT<0,9mm or plaque)	370	REGICOR model 5adapted FRS for Spain, factor 1,5 for RA, AUROC 0,74 Underestimates high risk patients, failed to identify >75% of ultrasound lesions	N/A	N/A	N/A	SCORE model, factor 1,5 for RA AUC= 0,79 Underestimates high risk patients, failed to identify >75% of ultrasound lesions	N/A	N/A
Willers [**20**]	2011	Cross sectional		100	N/A	N/A	N/A	N/A	Seems to underestimate risk even if the EULAR multiplier is used systematically comparative to another RA population [**21**]	N/A	N/A
Gomez-Vaquero [**22**]	2012	Cross sectional	N/A	200	N/A	N/A	N/A	N/A	Comparison between *mSCORE* and *SCORE*. No statistical difference in reclassification using *mSCORE* but high prevalence of CVD identified. *mSCORE* possibly fails to reclassify intermediate risk patients in higher groups	N/A	N/A
Arts [**23**]	2014	Cross sectional	CVD		Underestimates high risk	N/A	N/A	N/A	Underestimates high risk	Underestimates high risk	Underestimates global risk

**Risk - factor variables used in GP models**

All the models were based on a combination of traditional risk factors. Of those, age, gender, smoking status, total cholesterol levels and systolic blood pressure were constant among models. 3 models incorporated CVD family history and 3 others ethnic and geographic considerations. One model (QRisk2) incorporated 3 other comorbidity conditions of which, one was rheumatoid arthritis. PROCAM included triglyceride levels to the typical lipid profile. In contrast to the other models, SCORE and RSS models did not consider diabetes and previously treated hypertension as a separate risk factors. Two models incorporated novel variables - hsCRP in the RSS and carotid intima-media thickness (cIMT) in the ARIC - IMT model. Detailed data is available in **Table 2**.

**Table 2:** Variables used in the selected models

		Variables used						
		FRS L	ARIC	ARIC-IMT	PROCAM	SCORE	Raynolds	QRisk
Fixed Risk Factors	Age	X	X	X	X	X	X	X
	Gender	-	X	-	-	X	-	X
	Population	-	-	-	X	-	X	X
	Relevant family History	X	X	X	X	X	X	X
Modifiable Risk Factors	Smoking status	X	X	X	X	X	X	X
	Systolic blood pressure	X	X	X	X	X	X	X
	HDL Cholesterol level	X	X	X	X	X	X	X
	Total Cholesterol Level	X	X	X	X	X	X	X
	Body mass index	-		-	-	-	-	-
	Triglycerides			-	X	-	-	-
Ongoing Conditions	Current hypertension treatment	X	X	X	X	X	-	X
	Ongoing Diabetes	X	X	X	X	X	-	X
	Ongoing Rheumatoid arthritis	-		-	-	-	-	X
	Ongoing chronic Kideny disease	-		-	-	-	-	X
	Ongoing Artian Fibrillation	-		-	-	-	-	X
Emergent Risk Factors	hsCRP Level	-	-	-	-	-	X	-
Vascular imaging	Carotid intima-medica thickness	-	-	X	-	-	-	-
	Carotid Plaque	-	-	X	-	-	-	-

## Discussion

Probably because of its chronic, multisystem characteristics, even when suspected, RA CVD risk is often insufficiently addressed [**6**,**24**]. One of the expected explanation of this shortcoming is the lack of a standardized evaluation model.

In the GP all the models have similar - but not identical - moderate to good performances, easily explained by the use of algorithms based on the same traditional risk factors [**25**,**26**]. Two of the main differences derive rather from geographical or temporal reasons and from outcome definition (from soft to hard CVD events).

**Implementation in rheumatoid arthritis:**

Only two of the selected models (FRS and SCORE) were ever included in the international guidelines for CVD prevention in RA, according to the latest EULAR statement [**7**] and named mFRS and mSCORE.

In RA, the 4 models already validated proved to have overall moderate performances. In the US based study comparing FRS to SCORE, there is a clear tendency to underestimate cardiovascular risk, especially in the low and intermediate risk groups and overestimate risk in the high-risk group when the EULAR multiplier is applied. This is again demonstrated in another cohort study based in the Netherlands in which FRS, SCORE, RRS underestimated CVD risk especially in the low risk groups, this result not being noted for QRisk2. QRisk2 has the particularity that it is calibrated to include rheumatoid arthritis. It seems to overestimate the CVD risk. Since the majority of patients and CVD events are found in the low and intermediate risk groups, the usefulness of such empirical application of GP studies in RA population might not be appropriate.

In agreement with these results are the findings of the 3 cross-sectional studies cited, although their methodology has serious limitations (using surrogate markers over actual CVD or comparing the actual (mSCORE) model performance with results from a totally different cohort) (**Table 3**).

**Variables**

The small differences in performance for the already tested models could be explained by the existence of a common set of variables on which each algorithm is based. It is known that in the GP, traditional risk factors represent in fact those factors tied to the largest predictive effect. The introduction of non-traditional risk factors induces significant but small reclassification benefices. Their implementation in models for the GP is not recommended [**27**]. In contrast with the GP, it is accepted that in RA, the traditional RF does not explain for all the CVD and thus, regardless of the model notoriety, in our opinion, the change of attitude that would allow a better risk stratification in rheumatoid arthritis could be a progressive introduction of novel risk factors.

Two of the selected models are of interest, the RRS and ARIC-IMT. The odels accounting for the inflammation will be discussed further on. We pointed out that the only model (ARIC-IMT) uses the morphological assessments (carotid intima media thickness) directly, in addition to the traditional risk factor. The cIMT measurements proved to be of limited albeit statistically significant utility in the GP (GP) but its particular importance in rheumatoid arthritis will be addressed later on.

**Traditional risk factors (variables) which require a special attention in rheumatoid arthritis:**

**Age of onset of the disease:**

Age is an important predictor of CVD mortality in the GP and it has a central role in establishing the models previously described. Most models include patients over 40 years old but it should be reminded that for RA the age of onset can be sensibly lower [**28**]. Evidence points out that CVD events are more often seen early (within the first 7 years of the disease onset) and mortality is higher in the elderly [**29**,**30**]. The excess mortality seems to be explained by initially presenting the traditional risk factors but the early age onset of the disease is also linked to a higher incidence of traditional CVD RFs like hypertension [**31**]. It is not clear whether in RA age should be treated like a linear variable.

**BMI - body weight**

Due to chronic inflammation, cachexia is highly prevalent in RA. In contrast to the GP, there is evidence that a high body mass index is linked to a lesser disease burden [**32**]. BMI linked to CVD events is rather as dichotomous [**33**] than continuous and addressing CVD risk by using classical BMI variables that could lead to the over-evaluation of the CVD risk. Consistently, we excluded all the models by using BMI as a surrogate for lipid abnormalities in the GP. One variable that could be evaluated in RA CVD prediction models is the waist to hip ratio.

**Lipid profile and metabolic syndrome**

If the GP lipid abnormalities are directly linked to an increased cardiovascular risk, this is not true for the RA population. CVD risk was not increased with total cholesterol levels over 4mmol but rather inversely, it rose with the decrease of the total cholesterol. LDL cholesterol levels over 2mmol/l were only modestly linked to an increase of CVD risk and the risk augmented inversely to LDL levels [**34**]. The culprit for these anomalies is thought to be the systemic inflammation [**35**] and the effective treatment normalizes the lipid profile. The RA lipid profile is marked by a high triglyceride, high small dense LDL cholesterol and low HDL Cholesterol levels higher [**36**]. The total cholesterol and LDL cholesterol are included in every previously described model of CVD prediction and even when these controversies are well known, evidence exists that not all the authors utilize this knowledge accordingly [**37**].

The metabolic syndrome (MS) as defined by the ATPIII/IDF is clearly associated with higher CVD risk in the GP. In RA, MS has a similar or lower prevalence than in the GP [**38**] and seems to be linked to the presence of RF or ACPA [**39**]. Since both RF and ACPA are severity surrogate biomarkers, it can be expected that MS is linked to a more severe disease. Unexpectedly, no clear link to GC treatment was proven [**40**].

Their use, especially of the lipid profile, in future models should probably be adapted to the RA “lipid paradox” and controlled for inflammation and disease activity co variation.

**Novel risk factors to be considered in rheumatoid arthritis specific models:**

**Inflammation**

Chronic inflammation (regardless of RA disease activity) is positively linked to CVD risk. the average CRP levels over 12mg/dl in RA patients were independently tied to a 3 fold risk for CVD [**41**]. The Raynolds Risk Score, the model using hsCRP as a risk factor, has a much lower threshold for CRP than the average RA patient [**42**], so it might overestimate CVD risk in controlled disease and reversely, the underestimation of its inflammation being persistent. In multiple studies both the ESR and CRP levels were associated with the cardiovascular events [**43**] and especially with mortality [**44**].

**Disease activity and severity, FR and ACPAs**

Multiple evidence tie disease activity, severity and seropositivity (FR or ACPA) to poorer disease specific and CVD outcomes [**43**,**45**]. A higher DAS is linked to CVD independently of the traditional risk factors [**45**-**48**]. Even when disease activity is measured with the clinical Disease activity index, an office based score that excludes inflammatory lab variables, a higher disease activity is linked to CVD risk [**49**].

**The treatment related risk factors**

Both NSAIDs and corticosteroids have been tied to increase in CVD risk. The use of NSAIDs in RA patients is linked in a dose-dependent manner to CVD risk [**50**,**51**]. Corticosteroids are reported (although inconsistently [**41**]) to increase the risk of CVD especially in elderly patients [**45**] and in a dose-dependent manner [**52**], starting from a daily minimum dose of 8mg of methylprednisolone or a total dose of 40 grams. Prevalence of CVD risk factors is higher in prednisone treated patients [**53**]. On the contrary, the early use of DMARDs and especially biological agents seems to positively influence CVD outcomes of the RA patient, once again with a more visible effect in the elderly [**54**]. Although the use of corticoid sparing regimens and early use of biological agent is advocated [**7**], major differences between treatment habits and availability could probably result in differences in exposure to RA specific risk factors. A more severe disease could justify the need for corticosteroid for longer periods.

**Deprivation and cardio-respiratory fitness**

Compared with the GP, rheumatoid arthritis patients are sedentary and prone to social deprivation. None of the models presented here takes fitness into account. Almost half of the RA patients suffer from physical inactivity [**55**,**56**] and there is evidence that the improvement in cardiorespiratory fitness is linked to the improvement in the overall CVD risk factors [**57**]. The economic status is a major determinant of CVD prevalence [**6**,**58**], both in the GP and in RA. Coexistence with a poor socioeconomic status and RA leads to insufficient care [**59**]. Of the selected models, only one addresses deprivation in particular, not specifically to RA but rather to geographical origin (Qrisk2).

**Special consideration**

The need for morphological measurement is of special interest. It has been now recognized that one overlooked component of the extra-articular involvement of RA is rheumatoid vasculitis and it participates to early atherosclerosis [**60**]. Vasculitis and early atherosclerosis can be assessed by a number of imaging techniques status, but if PET CT is laborious and costly [**61**], cIMT and plaque measurements are easily available in common practice and seem to address well the particularities of the RA arteries [**30**,**62**,**63**] and outperform coronary arteries calcification scores [**64**].

## Conclusion

Current evidence suggests that addressing CVD risk in rheumatoid arthritis with GP models of prediction is of limited interest. New models of CVD risk prediction incorporating RA specific variables or imaging techniques are needed.

**Supplementary data**

PubMed search algorithm: multistep - (“cardiovascular” OR “coronary heart disease”) AND “risk” AND (“calculator” OR “model” OR “score” OR “prediction”) AND (“cardiovascular” OR “coronary heart disease”) AND “risk” AND (“calculator” OR “model” OR “score” OR “prediction”) AND “rheumatoid arthritis”)). The search term “validation”.

Models excluded: Framingham 1991, FRS1998, FRS 2008, WHO/IHS, JBS, ASSIGN, SCORE office based, PROCAM office based, FRS office based, Raynolds symplified, UKPDS, ADVANCE, DCS, NDR, Freemantle, CHS, ARIC (Folsom), HKDR, EPIC (NL and Potsdam), SMART, EPIC Norfolk, INTERHEART, AusSCORE, LIPID, Tayside, Australia Avso.
